# Cleft lip and palate risk factors among otorhinolaryngology: Head and neck surgery patients in two hospitals

**DOI:** 10.1097/MD.0000000000034419

**Published:** 2023-10-20

**Authors:** Louei Darjazini Nahas, Mariam Hmadieh, Mayssam Audeh, Abdulmajeed Yousfan, Imad Addin Almasri, Nafiza Martini

**Affiliations:** a Department of Surgery, Division of Otorhinolaryngology, Faculty of Medicine, Syrian Private University, Damascus, Syrian Arab Republic; b Syrian Private University, Faculty of Medicine, Damascus, Syrian Arab Republic; c Damascus University, Faculty of Medicine, Damascus, Syrian Arab Republic; d Damascus University, Faculty of Economics, Statistics Department, Damascus, Syrian Arab Republic; e Stemosis for Scientific Research, Damascus, Syrian Arab Republic.

**Keywords:** cleft lip and palate, consanguinity, family history, folic acid, malformation, medical history, risk factors

## Abstract

Cleft lip and/or palate is the most prevalent type of head and neck deformity, accounting for 65% of cases. The occurrence of this condition is influenced by both genetic and environmental factors. Cleft defects are classified into 2 types: syndromic cleft lip and palate syndrome and non-syndromic cleft lip and palate syndrome. Cleft lip with or without cleft palate is the most common type of cleft defect, and the surgical repair is the primary treatment option for patients. Our study was a retrospective case–control study that included 132 cases of patients with cleft defects and 132 healthy babies without cleft defects serving as controls. Personal information, including the name, age, and origin of the participants, was collected. Additionally, we collected information on all potential risk factors, including medical history, daily habits, consanguinity between parents, and family history. Information was collected in Excel and analyzed using the Statistical Package for Social Sciences and a Chi-Square test was performed to determine the results and their relationship to cleft lip and palate. Our study identified various risk factors that have a significant association with cleft lip and palate with a *P*-value <5% in addition to factors that are not considered risk factors. Using relative risk analysis, we were able to rank the top 5 most significant and influential risk factors. The most impactful factor was not taking folic acid during pregnancy. The primary risk factors associated with cleft lip and palate include a family history of the condition, lack of folic acid supplementation, maternal age over 35 years, and high temperatures exceeding 39 °C. Consequently, we recommend that mothers who intend to conceive should take folic acid supplements at a dose of 0.4 to 0.8 mg during the initial trimester of pregnancy. Additionally, we advise careful monitoring of all risk factors, particularly during the first trimester of pregnancy.

## 1. Introduction

Congenital head and neck malformations are among the most prevalent genetic malformations, with cleft lip with or without cleft palate being the most common, accounting for 65% of head and neck malformations. Cleft lip with or without cleft palate occurs due to abnormal embryonic development of the lip and palate.^[1]^ The incidence of cleft defects worldwide is estimated to be 1 in 700 live births,^[2]^ with higher rates observed among Native Americans and Asians (1 in 500 births) compared to Africans (1 in 2500 births).^[3]^

The occurrence of cleft defects is influenced by various genetic and environmental factors, including alcohol, smoking, teratogenic drugs, injury, diabetes, lack of folic acid intake, and high fever during pregnancy.^[4]^ Additionally, a family history of cleft defects and endogamy have been identified as risk factors for cleft defects.^[5]^

The cleft is classified into clefts with the syndrome and isolated clefts without the syndrome.^[6]^ The cleft not associated with a syndrome has a common cause between environmental and genetic factors, and it constitutes 1.25 per 1000 live births^[7]^ and the most common malformation associated with them is cardiac malformations.^[8]^

Cleft defects associated with syndromes are primarily caused by genetic factors and occur in specific syndromes such as Pierre Robin, Golden Har, Patu, Edwards, and van der Woude syndromes. These syndromes are characterized by cleft defects as well as other organ malformations,^[9]^ and the most common syndrome associated with clefts is Pierre Robin.^[10]^

Cleft defects are classified into 3 main types: isolated cleft lip, isolated cleft palate, and cleft lip with palate. Cleft lip is further divided into unilateral and bilateral clefts, as well as complete and incomplete clefts, based on the degree of extension of the cleft to the nasal floor.^[4]^ The cleft palate is also divided into unilateral, bilateral, complete, incomplete, and submucosal.^[11]^

The most common type of injury is cleft lip and palate.^[4]^ A cleft lip is more common in males than females in a ratio of 2:1, and also cleft palate is more common in females than males.^[4]^ Individuals with cleft defects may experience various psychological, social, pathological, and aesthetic challenges, as well as speech and feeding problems, particularly those with cleft palate. They are also at a higher risk of developing otitis media and dental problems.^[6]^ Surgical repair is the primary treatment for patients with cleft, and it will radically change the patient’s life^[4]^

We aimed in this study to define and assess primary risk factors associated with cleft lip and palate, such as kinship marriage, folic acid intake, fever, and others.

## 2. Materials and methods

### 2.1. Study design

The pattern of our study is a retrospective study (case–control study), and information was collected from hospital archives.

### 2.2. Study subject

For our study, we collected a sample of cases from the archives of various hospital departments, including ear, nose and throat, maxillofacial surgery, pediatric surgery, and plastic surgery departments. We conducted our study in 2 hospitals, Damascus Hospital and Al-Mowasat Hospital, between 2016 and 2022.

In addition to collecting data from hospital archives, we also contacted the families of patients to gather additional information through the phone numbers provided in the files. We obtained informed consent from participants before collecting any personal information. The final sample size for our study was 264 participants, consisting of 132 cases and 132 controls.

### 2.3. Data collection

We entered the collected data from our sample of patients into an Excel spreadsheet, classifying each variable as either qualitative, quantitative, or numerical (sex, male, and female). We documented the presence of cleft defects in our sample of patients, noting whether the patient had an isolated cleft lip, isolated cleft palate, or cleft lip and palate. Additionally, we classified the cleft lip and palate based on its direction and extent.

We included questions in our study regarding various potential risk factors that the mother may have been exposed to during pregnancy, such as hypertension, diabetes, smoking, alcohol consumption, maternal age during pregnancy, medication use, and medical history, consanguinity between parents, family history, high fever during pregnancy, exposure to radiation during pregnancy, and maternal blood group. We also collected information on any accompanying malformations or syndromes in the child, as well as the child’s incidence of middle ear inflammation.

### 2.4. Data analysis

The statistical tests that fit the research data were used, which are the nominal and ordinal data, as most data were reported as numbers and ratios. A Chi-Square test was used to assess the correlation between groups and each variable related to the cleft lip and palate for all purposes, and relative risk factors were calculated to expecting the probability of the injury and arranging the most significant risk factors.

Also, using Mann–Whitney test, we compared between the mothers ages.

statistical significance was determined as a *P* < .05. All analyses were performed using the Statistical Package for Social Sciences (SPSS-25).

### 2.5. Sample size

The sample size (n) was determined by Cochran sample size formula with the assumption of 95.5% confidence level (*Z* = 2), *e* is the margin of error which is 10%, *p* is the (estimated) proportion of the population which has the attribute in question, and it equals 50% (or 0.5), and *q* is 1 − *p*


$$\begin{array}{l} n=\frac{z^{2}pq}{e^{2}}=\frac{{\left(2\right)}^{2}*0.5*0.5}{{\left(0.1\right)}^{2}}=100 \end{array}$$

The required sample size (n) for this study, applying the previous formula, is at least 100.

If we adopt a 5% margin of error. The sample size becomes 400.


$$\begin{array}{l} n=\frac{z^{2}pq}{e^{2}}=\frac{{\left(2\right)}^{2}*0.5*0.5}{{\left(0.05\right)}^{2}}=400 \end{array}$$

Therefore, with a confidence of 95.5 and an error ranging from 5% to 10%, a sample size ranging from 100 to 400 individuals should be required. And we collected 264 participants.

We had sought to have relied on the extent of the error of 5%, so depending on the previous equation, we must collect 400 questionnaires. However, after a full month of the collection process, due to the difficulties we faced, we did not reach more than 264 participants.

### 2.6. Ethics approval and consent to participate

The Ethical Committee approved this study in the Faculty of Medicine Syrian Private University, Syria with a number (356). The study complied with the principles of the Helsinki Declaration.

## 3. Results

After analyzing the data and information, the results of our study were as follows:

Endogamy and family history were associated with cleft lip/palate with a rate of 56/132 in cases compared to 39/132 in controls and 44/132 in cases compared to 1/132 in controls with significance level (sig) of .029 and <.05, respectively (Table 1).

**Table 1:** Crosstab.

	Groups		Total	*P*-value
	Cases (patients)	Controls (intact)		
Consanguinity between the mother and the father				
There is kinshipnot	76(45.0%)	93(55.0%)	**169(100%**)	.05 > .029*
There is kinship	56(58.9%)	39(41.1%)	**95(100%**)	
Total	**132(50.0%**)	**132(50.0%**)	**264(100%**)	
Having a family history				
Not having	88(40.2%)	131(59.8%)	**219(100%**)	.05 > .000*
Have	44(97.8%)	1(2.2%)	**45(100%**)	
Total	**132(50.0%**)	**132(50.0%**)	**264(100%**)	
First degree consanguinity				
No	114(46.3%)	132(53.7%)	**246(100%**)	.05 > .000*
Yes	18(100.0%)	0(0.0%)	**18(100%**)	
Total	**132(50.0%**)	**132(50.0%**)	**264(100%**)	
Second degree consanguinity				
No	121(47.8%)	132(52.2%)	**253(100%**)	.05 > .001*
Yes	11(100.0%)	0(0.0%)	**11(100%**)	
Total	**132(50.0%**)	**132(50.0%**)	**264(100%**)	
Third degree consanguinity				
No	114(46.5%)	131(53.5%)	**245(100%**)	.05 > .000*
Yes	18(94.7%)	1(5.3%)	**19(100%**)	
Total	**132(50.0%**)	**132(50.0%**)	**264(100%**)	
Diabetes injury				
No	127(49.0%)	132(50.1%)	**259(100%**)	.05 > .024*
Yes	5(100.0%)	0(0.0%)	**5(100%**)	
Total	**132(50.0%**)	**132(50.0%**)	**264(100%**)	
Severe fever > 39°				
No	80(39.4%)	123(60.6%)	**203(100%**)	.05 > .000*
Yes	52(85.2%)	9(14.8%)	**61(100%**)	
Total	**132(50.0%**)	**132(50.0%**)	**264(100%**)	
X-rays during pregnancy				
No	128(49.2%)	132(50.8%)	**260(100%**)	.05 > .044*
Yes	4(100.0%)	0(0.0%)	**44(100%**)	
Total	**132(50.0%**)	**132(50.0%**)	**264(100%**)	

* means (significant).

Our study found that the most common gestational age was 37 weeks and the most common age group in the cases is between 20 to 24 with rate 45/132 of the cases. Also, our study revealed a significant association between the incidence of cleft defects and the age groups of mothers, with mothers aged 35 years and above being at higher risk with sig values <.05 (Table 2).

**Table 2:** Crosstab.

	Groups		Total	*P*-value
	Cases (patients)	Controls (intact)		
Presence of serous otitis media in the child				
No	63(32.8%)	129(67.2%)	**192(100%**)	.05 > .000*
Yes	69(95.8%)	3(4.2%)	**72(100%**)	
Total	**132(50.0%**)	**132(50.0%**)	**264(100%**)	
Intake folic acid				
Intake	23(23.2%)	76(76.8%)	**99(100%**)	.05 > .000*
Not intake	109(66.1%)	56(33.9%)	**165(100%**)	
Total	**132(50.0%**)	**132(50.0%**)	**264(100%**)	
Intake corticosteroids (pregnancy stabilizer)				
No	107(54.6%)	89(45.4%)	**196(100%**)	.05 > .011*
Yes	25(36.8%)	43(63.2%)	**68(100%**)	
Total	**132(50.0%**)	**132(50.0%**)	**264(100%**)	
Intake antibiotics				
No	111(47.2%)	124(52.8%)	**235(100%**)	.05 > .011*
Yes	21(72.4%)	8(27.6%)	**29(100%**)	
Total	**132(50.0%**)	**132(50.0%**)	**264(100%**)	
Analgesic				
No	115(46.6%)	132(53.4%)	**247(100%**)	.05 > .000*
Yes	17(100.0%)	0(0.0%)	**17(100%**)	
Total	**132(50.0%**)	**132(50.0%**)	**264(100%**)	
Head and neck malformation				
No	125(48.6%)	132(51.4%)	**257(100%**)	.05 > .007*
Yes	7(100.0%)	0(0.0%)	**7(100%**)	
Total	**132(50.0%**)	**132(50.0%**)	**264(100%**)	
Urinary tract				
No	124(48.4%)	132(51.6%)	**256(100%**)	.05 > .004*
Yes	8(100.0%)	0(0.0%)	**8(100%**)	
Total	**132(50.0%**)	**132(50.0%**)	**264(100%**)	
Heart disease (such as Ventricular septal defect)				
No	127(49.0%)	132(51.0%)	**259(100%**)	.05 > .024*
Yes	5(100.0%)	0(0.0%)	**5(100%**)	
Total	**132(50.0%**)	**132(50.0%**)	**264(100%**)	
Mother’s age during pregnancy				
−20	17(38.6%)	27(61.4%)	**44(100%**)	.05 > .037**
20–24	45(49.5%)	46(50.5%)	**91(100%**)	
25–29	26(46.4%)	30(53.6%)	**56(100%**)	
30–34	27(58.7%)	19(41.3%)	**46(100%**)	
35+	17(63.0%)	10(37.0%)	**27(100%**)	
Total	**132(50.0%**)	**132(50.0%**)	**264(100%**)	

* Pearson Chi-Square test sig.

** Mann–Whitney test sig.

We found only 2 cases that were syndromic cleft lip and palate syndrome, namely 1 case of Pierre Robin syndrome and 1 case of Goldenhar syndrome. There was a positive relationship between each (not taking folic acid sig = .00, taking steroids sig = .011, taking antibiotics sig = .011, high fever sig = .00) and the occurrence of cleft lip/palate (Table 2).

And we did not find a relationship between each of the daily habits (smoking and alcohol, taking anticonvulsants, and high blood pressure) and the incidence of cleft lip/palate with a value of sig > .05 (Table 3).

**Table 3:** The variable is not related with cleft lip.

Variable	Pearson Chi-Square sig.
Smoking “Hablpabol”	0.05 < 0.762
Hypertension injury	0.05 < 0.076
Intake anticonvulsants	0.05 < 0.316
Intake a retinoid acid (Retan)	0.05 < 0.316
Brain malformations	0.05 < 0.099
Bone malformations	0.05 < 0.156
Finger fusion	0.05 < 0.156
Thoracic malformations	0.05 < 0.409
Intestinal (cecum obstruction…. Hedratresia…)	0.05 < 0.316
Blood group of the mother	0.05 < 0.112

The most common type of clefts in our study was cleft lip and palate (CLP 70/132 [53%]) and isolated cleft palate (CPO 41/132 [31.10%]). Finally, isolated cleft lip (CLO 21/132 [15.90%]). The most common classification of the cleft lip was complete bilateral 37/132 (28%), and the least common was incomplete bilateral 5/132 (3.8%). As for the palate, the most common was incomplete unilateral 39/132 (29.50%), and the least common was the incomplete cleft lip bilateral 7/132 (5.3%) (Table 4).

**Table 4:** Crosstab

	Groups		Total
	Cases (patients)	Controls (intact)	
Gender			
Male	69(52.2%)	61(46.2%)	130(49.2%)
Female	63(47.7%)	71(53.7%)	134(50.7%)
Total	132(100%)	132(100%)	264(100%)

	Frequency	Valid percent	
Cleft palate			
Incomplete unilateral	39	29.5%	
Completely unilateral	31	23.5%	
Incomplete bilateral	7	5.3%	
Completely bilateral	34	25.8%	
Intact	21	15.9%	
Total	**132**	100%	
Injury pattern			
Isolated cleft lip	21	15.9%	
Isolated cleft palate	41	31.1%	
Cleft lip and palate	70	53.0%	
Total	**132**	100%	
Cleft lip			
Incomplete unilateral	19	14.4%	
Completely unilateral	30	22.7%	
Incomplete bilateral	5	3.8%	
Completely bilateral	37	28.0%	
Intact	41	31.1%	
Total	**132**	100%	

The percentage of males in the case group was 69/132 (52.27%) and the percentage of females was 63/132 (47.73%), while in the group of controls, the percentage of males was 61/132 (46.21%) and the percentage of females was 71/132 (53.79%) (Table 4).

### Relative risk factor.

The risk factor is considered a predictive indicator during studying the correlation using Chi-Square to know the most significant correlated risk factor for the occurrence of such clefts. Perspective factors to the occurrence of these malformations, and their arrangement from the most serious to the least dangerous.

The risk of cleft lip or cleft palate or both, if a woman does not take folic acid, is 184% higher than the risk if she takes folic acid.

For having a family history, the probability of a child suffering from cleft lip, cleft palate or both is 143% higher than the probability of the injury in the absence of the risk factor (hereditary/family history).

The risk of cleft lip or palate if a woman had a high temperature of more than 39 degrees during pregnancy is 116% higher than the risk if the woman did not have a high temperature during pregnancy.

The probability of cleft lip or palate in case of taking antibiotics (medication history) for a severe fever of more than 39° is higher by 53.3% than the probability of the injury in the event of not taking antibiotics.

In the event that there is a consanguinity marriage, the probability of the child suffering from cleft lip, cleft palate or both is higher by 31.1% than the probability of the injury in the absence of the risk factor of the consanguinity marriage between the father and the mother (Table 5).

**Table 5:** Risk estimate.

Variable	Value	95% confidence interval		
		Upper	Lower	
Woman does not take folic acid	2.843	1.955	4.135	1st
Having a family history	2.433	2.058	2.877	2nd
High temperature	2.163	1.771	2.642	3rd
Taking antibiotics	1.533	1.180	1.993	4th
Kinship marriage	1.311	1.035	1.661	5th

The results of Table 6 show the multiple logistic regression of the risk factors associated with the incidence, and the results indicate that the model is appropriate, as the *P*-value (model) is less than the significance level of .05. Therefore, at least one (or all) of the factors is significant for logistic regression. The *R*-square value indicates that 73% of the variations in the occurrence or nonoccurrence of the disease can be explained by the model. Based on this, it is shown that:

**Table 6:** Multivariate analysis.

	B	OR (EXP(B))	*P*-value	95% C.I.for EXP(B)	
				Lower	Upper
Woman does not take folic acid	2.104	8.201	<.001*	3.438	19.560
Having a family history	3.947	5.780	<.001*	2.650	12.502
High temperature	1.660	5.259	<.001*	1.672	9.349
Taking antibiotics	0.986	2.680	<.001*	0.877	8.186
Kinship marriage	0.517	1.678	<.001*	0.868	3.242

*R*-square = 0.73.

* *P*-value (model) < .001.

- The factor represented by maternal intake of folic acid is statistically significant at the significance level of .05, and its significance can be interpreted as follows: every transition from the mother taking folic acid to not taking it leads to an increased likelihood of the transition from the child not being born with cleft lip and palate to being born with it by a factor of 8.2.
- The factor represented by family history is statistically significant at the significance level of .05, and its significance can be interpreted as follows: every transition from the absence of a family history to its presence leads to an increased likelihood of the transition from the child not being born with cleft lip and palate to being born with it by a factor of 5.78.
- The factor represented by severe high temperature is statistically significant at the significance level of .05, and its significance can be interpreted as follows: every transition from severe high temperature to a higher temperature leads to an increased likelihood of the transition from the child not being born with cleft lip and palate to being born with it by a factor of 5.25.
- The factors represented by antibiotic use and consanguineous marriage are also statistically significant at the significance level of .05.

## 4. Discussion

Congenital cleft lip and/or palate is a significant and widespread condition, with a prevalence rate of approximately 1 in 700 children globally. Its importance stems not only from its high incidence but also from the psychological and social impact it can have on the affected child, their family, and society as a whole.

Upon examination of all cases and types of defects, it was observed that 69 out of 132 (52.27%) of those affected were male and 63 out of 132 (47.73%) were female. Similar gender distribution with higher male dominance was observed in a study in Saudi Arabia^[7]^ at 81 out of 130 (62%), while female majority at 82 out of 150 (54.7%) was found in a Nigerian study.^[12]^

Cleft lip and palate 70 out of 132 (53%) represented the most frequent type of cleft defects, which is similar to previous studies.^[1,13]^ Moreover, the second most frequent type in our study was cleft palate followed by cleft lip (see Table 4), while vice versa was reported in previous studies.^[1,13]^

In our study, complete bilateral cleft lip was the most common type of cleft lip, accounting for 37 out of 132 (28%) of cases, followed by complete unilateral cleft lip at 30 out of 132 (22.7%), incomplete unilateral cleft lip at 19 out of 132 (14.4%), and incomplete bilateral cleft lip at 5 out of 132 (3.8%) (see Table 4). By comparison, in a previous study conducted in Colombia, the estimated rate of unilateral cleft lip was 11.4% (236 out of 2069), while the rate of bilateral cleft lip was 2.08% (43 out of 2069).^[14]^ In a study carried out in the Kingdom of Saudi Arabia, the estimated rate of unilateral cleft lip was 20% (26 out of 130), while the rate of bilateral cleft lip was 5% (7 out of 130).^[7]^

In our study, for cleft palate, incomplete unilateral cleft palate was the most common with a rate of 39 out of 132 (29.5%), followed by complete bilateral 34 out of 132 (25.80%), then complete unilateral 31 out of 132 (23.50%), and finally Incomplete Bilateral 7 out of 132 (5.30%) (Table 4). In comparison to a previous study conducted in the Kingdom of Saudi Arabia,^[7]^ the number of patients with unilateral cleft palate was 14 out of 130 with an estimated rate of 11%, while bilateral was 11 out of 130 with an estimated rate of 8%.^[7]^ In another study conducted in Peru,^[15]^ cleft palate was unilateral (309 out of 3923), while bilateral was (154 out of 3923).^[15]^

Our findings that mothers aged 35 or more are at higher risk of cleft palate go in line with a previous study.^[5]^ In contrast, a Nigerian study^[16]^ showed age <25 was associated with a higher risk of having a child with a cleft defect. But also highlighted mother age over 39 to have an increased risk of having a child with cleft defects. Our study found that the most common gestational age was 37 weeks, which is consistent with a study conducted in Mexico in 2017. In that study, the average gestational age was also reported to be 37 weeks.^[17]^

Similar to a Chinese study in 2021,^[5]^ our findings showed statistically significant association between consanguinity between the father and mother and the incidence of cleft lip, cleft palate, or both defects combined. However, this relationship was not observed in a study in 2022 from Saudi Arabia.^[6]^ Also, previous studies from Brazil^[18]^ and Saudi Arabia^[6]^ indicated a significant relationship between the incidence of cleft defects and family history, which is noted in our study. First-degree and second-degree family history of clefts were found to be at higher risk than third degree. In addition, our study and an Indian study in 2017^[19]^ did not observe any association between cleft defects and diabetes or high blood pressure.

Maternal diabetes and cleft defect were found to be associated in our study as well as in a Romanian study^[20]^ that reported diabetic mothers are likely to have children with cleft defects 3 times more than healthy mothers. Moreover, maternal smoking and alcohol consumption during pregnancy are not associated with cleft defects, which agrees with a study conducted in Vietnam.^[21]^ However, we found a significant relationship between not taking folic acid (sig = .00), taking steroids (sig = .001), and taking antibiotics (sig = .01) during pregnancy and the incidence of cleft defects. These findings are consistent with the results of other studies.^[22,23]^

Furthermore, a study in Brazil^[22]^ showed similar results to our study, indicating no association between anticonvulsants use during pregnancy and cleft defects (Table 3).

Our study shows a significant relationship between the use of analgesics during pregnancy and the incidence of cleft defects. However, we did not find any previous studies that reported similar findings. Interestingly, a study conducted in Brazil reported that taking analgesics during pregnancy is a protective factor against cleft defects.^[22]^ Our results are in line with a previous study^[24]^ that noted the important relationship between maternal high fever during pregnancy and cleft defects. Our results present a weak relationship between exposure to radiation and the incidence of cleft defects, which is similar to a previous study from Brazil.^[22]^ Additionally, a 2017 study in Iran^[25]^ observed that all malformations were remarkably high for cleft patients, which is consistent with our results that indicate other malformations in children, such as head and neck malformations, urinary system issues, and heart diseases like ventricular septal defect, are associated with cleft lip, palate, or both.

Sometimes, cleft defects are associated with other syndromes, as shown in a 2021 study^[26]^ where cleft accompanied by syndromes represented 24 out of 266 cases. However, our results showed that only 2 out of 132 patients had cleft defects associated with syndromes.

Otitis media was found to be associated with cleft defects in a previous study^[25]^ as well as in our study, where 69 out of 132 cases had otitis media compared to only 3 out of 132 controls.

## 5. Limitations

Due to the chaotic situation during COVID-19 and war time in Syrian Arab Republic, there was not enough information in the hospital’s archives. Additionally, some telephone numbers in the archives were incorrect, making it challenging to contact patients and gather additional information.

## 6. Conclusion

The primary risk factors associated with cleft lip and palate include a family history of the condition, lack of folic acid supplementation, maternal age over 35 years, and high temperatures exceeding 39 °C. Consequently, we recommend that mothers who intend to conceive should take folic acid supplements at a dose of 0.4 to 0.8 mg during the initial trimester of pregnancy. Additionally, we advise careful monitoring of all risk factors, particularly during the first trimester of pregnancy.

## Acknowledgments

We wish to show our appreciation to Stemosis for Scientific Research, a Syria-based scientific research youth team managed by Nafiza Martin**i**, for the scientific environment they provided. Also, we wish to thank members from Stemosis for Scientific Research, Dr Majd Hanaa, Mhd Moumen Almouallem, Dr Yara Ghazi, and Dr Aya Al Maghout, for their help in English checking and reviewing this paper.

## Author contributions

**Conceptualization:** Louei Darjazini Nahas, Mariam Hmadieh, Mayssam Audeh, Abdulmajeed Yousfan, Nafiza Martini.

**Data curation:** Imad Addin Almasri.

**Formal analysis:** Imad Addin Almasri.

**Methodology:** Mariam Hmadieh, Mayssam Audeh, Imad Addin Almasri.

**Supervision:** Louei Darjazini Nahas, Abdulmajeed Yousfan, Nafiza Martini.

**Writing – original draft:** Mariam Hmadieh, Mayssam Audeh, Nafiza Martini.

**Writing – review & editing:** Louei Darjazini Nahas, Mariam Hmadieh, Mayssam Audeh, Abdulmajeed Yousfan, Imad Addin Almasri, Nafiza Martini.

## References

[1] Turlapati S Krishna S Deepak KU. A cross-sectional study: are myths on cleft lip and palate still prevalent? Cureus. 2021;13:e19579. 34926050 10.7759/cureus.19579 PMC8671751

[2] Fell M Dack K Chummun S. Maternal cigarette smoking and cleft lip and palate: a systematic review and meta-analysis. Cleft Palate Craniofac J. 2022;59:1185–200. 34569861 10.1177/10556656211040015 PMC9411693

[3] Nwaze CE Adebayo O Adeoye AM. Orofacial clefts and cardiovascular risk and diseases: the causal relationship and associations. Ann Ib Postgrad Med. 2020;18:S28–34. 33071693 PMC7513382

[4] Alois CI Ruotolo RA. An overview of cleft lip and palate. JAAPA. 2020;33:17–20. 10.1097/01.JAA.0000721644.06681.06 33234889

[5] Ahmed Sakran K Mutahar Abotaleb B Khaled Al-Rokhami R. Analysis of environmental exposures for nonsyndromic cleft lip and/or palate: a case–control study. Iran J Public Health. 2022;51:578–86. 35865051 10.18502/ijph.v51i3.8934 PMC9276608

[6] Alyami B Ali-Hassan M Al-Mahri M. Risk indicators for syndromic and nonsyndromic orofacial clefts in Southern Province of Saudi Arabia. J Cleft Lip Palate Craniofac Anomal. 2019;6:51–5.

[7] AlHammad Z Suliman I Alotaibi S. The prevalence of non-syndromic orofacial clefts and associated congenital heart diseases of a tertiary hospital in Riyadh, Saudi Arabia. Saudi Dent J. 2021;33:137–42. 33679106 10.1016/j.sdentj.2019.12.002 PMC7910677

[8] Toubat O Mallios DN Munabi NCO. Clinical importance of concomitant cleft lip/palate in the surgical management of patients with congenital heart disease. World J Pediatr Congenit Heart Surg. 2021;12:35–42. 33407037 10.1177/2150135120954814 PMC8138941

[9] Bui AH Ayub A Ahmed MK. Maternal tobacco exposure and development of orofacial clefts in the child: a case-control study conducted in Pakistan. Ann Plast Surg. 2018;81:708–14. 30300227 10.1097/SAP.0000000000001665

[10] Kumar PP S Dhull K G L. Incidence and demographic patterns of orofacial clefts in Mysuru, Karnataka, India: a hospital-based study. Int J Clin Pediatr Dent. 2018;11:371–4. 30787548 10.5005/jp-journals-10005-1542 PMC6379536

[11] Bekele KK Ekanem PE Meberate B. Anatomical patterns of cleft lip and palate deformities among neonates in Mekelle, Tigray, Ethiopia; the implication of environmental impact. BMC Pediatr. 2019;19:254. 31340768 10.1186/s12887-019-1624-2 PMC6657112

[12] Erinoso OA James O Sokunbi OJ. Congenital heart defects in orofacial cleft: a prospective cohort study. Afr J Paediatr Surg. 2021;18:219–23. 34341307 10.4103/ajps.AJPS_159_20 PMC8423164

[13] Eshete M. Pattern of orofacial clefts at a tertiary care hospital in Ethiopia. Ethiop J Health Sci. 2021;31:1175–84. 35392346 10.4314/ejhs.v31i6.12 PMC8968373

[14] Espinosa AS Martinez JC Molina Y. Clinical and descriptive study of orofacial clefts in Colombia: 2069 patients from operation smile foundation. Cleft Palate Craniofac J. 2022;59:200–8. 33736479 10.1177/10556656211000551 PMC8750128

[15] Peña-Soto C Arriola-Guillén LE Díaz-Suyo A. Clinical and epidemiological profile of cleft lip and palate patients in Peru, 2006–2019. J Clin Exp Dent. 2021;13:e1118–23. 34824698 10.4317/jced.58976 PMC8601701

[16] James O Erinoso OA Ogunlewe AO. Parental age and the risk of cleft lip and palate in a Nigerian population – a case–control study. Ann Maxillofac Surg. 2020;10:429–33. 33708590 10.4103/ams.ams_134_20 PMC7944012

[17] Angulo-Castro E Acosta-Alfaro LF Guadron-Llanos AM. Maternal risk factors associated with the development of cleft lip and cleft palate in Mexico: a case–control study. Iran J Otorhinolaryngol. 2017;29:189–95. 28819616 PMC5554809

[18] Maranhão SC Sá J Cangussú MCT. Nonsyndromic oral clefts and associated p- in the state *p*-value of Bahia a, Brazil. Eur Arch Paediatr Dent. 2021;22:121–7. 32274688 10.1007/s40368-020-00522-0

[19] Neogi SB Singh S Pallepogula DR. The p-value for orofacial clefts in India: a case–control study. Birth Defects Res. 2017;109:1284–91. 28766884 10.1002/bdr2.1073 PMC6686724

[20] Kozma A Radoi V Ursu R. Gestational diabetes mellitus and the development of cleft lip/ palate in newborns. Acta Endocrinol (Buchar). 2019;5:118–22. 31149070 10.4183/aeb.2019.118 PMC6535315

[21] Dien VHA McKinney CM Pisek A. Maternal exposures and risk of oral clefts in South Vietnam. Birth Defects Res. 2018;110:527–37. 29322637 10.1002/bdr2.1192 PMC6698255

[22] Regina Altoé S Borges AH Neves ATSC. Influence of parental exposure to *p*-value in the occurrence of oral clefts. J Dent (Shiraz). 2020;21:119–26. 32582827 10.30476/DENTJODS.2019.77620.0 PMC7280550

[23] Saarti M Mahmood MD Alchalaby LA. Overview of drug-induced orofacial cleft. Int J Pharm Pharm Stud (IJPPS). 2022;6:PP1-6. 38807406

[24] Waller DK Hashmi SS Hoyt AT. Maternal report of fever from cold or flu during early pregnancy and the risk for noncardiac birth defects, National Birth Defects Prevention Study, 1997–2011. Birth Defects Res. 2018;110:342–51. 29094488 10.1002/bdr2.1147 PMC5831519

[25] Jamilian A Sarkarat F Jafari M. Family history and risk factors for cleft lip and palate patients and their associated anomalies. Stomatologija. 2017;19:78–83. 29339670

[26] Bartzela T Theuerkauf B Reichardt E. Clinical characterization of 266 patients and family members with cleft lip and/or palate with associated malformations and syndromes. Clin Oral Investig. 2021;25:5531–40. 10.1007/s00784-021-03863-2 PMC8370934 33760974

